# Targeting Extracellular Vesicles to the Arthritic Joint Using a Damaged Cartilage-Specific Antibody

**DOI:** 10.3389/fimmu.2020.00010

**Published:** 2020-02-14

**Authors:** Louise M. Topping, Bethan L. Thomas, Hefin I. Rhys, Jordi L. Tremoleda, Martyn Foster, Michael Seed, Mathieu-Benoit Voisin, Chiara Vinci, Hannah L. Law, Mauro Perretti, Lucy V. Norling, Helena S. Azevedo, Ahuva Nissim

**Affiliations:** ^1^Barts and the London School of Medicine and Dentistry, William Harvey Research Institute, Queen Mary University of London, London, United Kingdom; ^2^Centre for Bioengineering, Life Sciences, Queen Mary University of London, London, United Kingdom; ^3^Barts and the London School of Medicine and Dentistry, Blizard Institute, Queen Mary University of London, London, United Kingdom; ^4^Experimental Pathology Consultancy, London, United Kingdom; ^5^School of Health Sport and Bioscience, University of East London, London, United Kingdom; ^6^Centre for Inflammation and Therapeutic Innovation, Queen Mary University of London, London, United Kingdom; ^7^School of Engineering and Materials Science, Institute of Bioengineering, Queen Mary University of London, London, United Kingdom

**Keywords:** rheumatoid arthritis, extracellular vesicles (EV), monoclonal antibodies, anti-TNF, collagen II

## Abstract

The targeted delivery of therapies to diseased tissues offers a safe opportunity to achieve optimal efficacy while limiting systemic exposure. These considerations apply to many disease indications but are especially relevant for rheumatoid arthritis (RA), as RA is a systemic autoimmune disease which affects multiple joints. We have identified an antibody that is specific to damaged arthritic cartilage (anti-ROS-CII) that can be used to deliver treatments specifically to arthritic joints, yielding augmented efficacy in experimental arthritis. In the current study, we demonstrate that scaffolds enriched with bioactive payloads can be delivered precisely to an inflamed joint and achieve superior efficacy outcomes consistent with the pharmacological properties of these payloads. As a scaffold, we have used extracellular vesicles (EVs) prepared from human neutrophils (PMNs), which possess intrinsic anti-inflammatory properties and the ability to penetrate inflamed arthritic cartilage. EV fortified with anti-ROS-CII (EV/anti-ROS-CII) retained anti-ROS-CII specificity and bound exclusively to the damaged cartilage. Following systemic administration, EV/anti-ROS-CII (a) exhibited the ability to localize specifically in the arthritic joint *in vivo* and (b) was able to specifically target single (viral IL-10 or anti-TNF) or combined (viral IL-10 and anti-TNF) anti-inflammatory treatments to the arthritic joint, which accelerated attenuation of clinical and synovial inflammation. Overall, this study demonstrates the attainability of targeting a pro-resolving biological scaffold to the arthritic joint. The potential of targeting scaffolds such as EV, nanoparticles, or a combination thereof alongside combined therapeutics is paramount for designing systemically administered broad-spectrum of anti-inflammatory treatments.

## Introduction

The development of anatomically targeted methods offers the promise for effective therapy localized at the site of disease, which optimizes pharmacological effect while minimizing systemic exposure and ensuring increased safety. Rheumatoid arthritis (RA) is the second most common form of arthritis in the world, characterized by long-term inflammation in the joints leading to cartilage and bone erosion and, eventually, joint deformation. In the context of RA, targeted approaches offer the promise of delivering highly effective disease-modifying agents to the affected joints without the limitations of systemic toxicity. Current therapies for the treatment of RA comprise synthetic or biologic disease-modifying antirheumatic drugs (DMARDs) (1). The development of small molecules and biologics has enabled some degree of disease modification in RA patients. Nevertheless, apart from a spectrum of adverse side effects, a significant proportion of patients (~40%) still have inadequate control of their arthritis activity and do not enter remission (2, 3). Thus, there remains a significant unmet need for improved treatment.

The current study investigates a novel form of drug targeting using extracellular vesicles (EVs) as a cargo to deliver single or multiple pharmacological payloads. Membrane-derived microparticles/microvesicles, apoptotic bodies, and exosomes are collectively known as EVs. EVs function in cell-to-cell communication and carry microRNA (miRNA), messenger RNA (mRNA), and hundreds of proteins and lipids (4–6). They transmit these cargoes to different cells to induce various changes in cell behavior, including transcription and proliferation (7–9). EVs vary in their contents, and in fact, the EV miRNA expression profile can serve as a potential biomarker (10, 11). EVs appear to play key roles in cancer progression and metastasis (12) and in the normal maintenance and degeneration of musculoskeletal tissues (13, 14).

An emerging approach of interest in the context of joint disease is the utilization of neutrophil (PMN)-derived EV to promote chondroprotective effects. In 2004, Gasser and Schifferli showed that PMN EV exhibited anti-inflammatory properties (15), and we reported that some of these are reliant to the presence of phosphatidylserine and annexin A1 (16, 17). EVs derived from PMN have been utilized as scaffolds for therapeutic purposes through loading with alpha-2-macroglobulin and an analogue of lipoxin A_4_ (18, 19). In a recent study, we uncovered the chondroprotective effects of PMN EV in the K/BxN serum transfer model of arthritis (20): these vesicles penetrated into arthritic cartilage tissue to promote anabolic activities yielding cartilage repair and protection (20). This concept has been extended by a more recent study, where the EV/PMN cell membrane was used to coat nanoparticles with reported significant therapeutic efficacy in a collagen-induced human transgenic mouse model of arthritis, with evident amelioration of joint damage and suppression of the overall arthritis severity (21). Importantly, all the above studies have been conducted with local administration of the microstructures (20), which places limitations on the effective translation of these findings into clinical settings. In the present study, we have used an antibody that is specific to damaged arthritic cartilage (anti-ROS-CII) to develop an effective preparation of EV that, upon systemic administration, specifically localizes EVs to the arthritic joint. As a proof of concept, we have also utilized these novel microstructures to deliver an anti-inflammatory payload—either anti-TNFα or viral IL-10 or both.

In recent years, the development of small molecules and biologics has greatly improved the treatment of RA; however, a significant proportion of patients (~40%) still have inadequate control of their arthritis activity and do not enter remission. Thus, there remains a significant unmet need for novel and improved therapeutic strategies.

## Materials and Methods

### Production of scFv and Fusion Antibodies

ScFv was produced as described (22). Transient transfection of IgG and vIL-10 fusion antibodies was conducted in an Expi293F system (Thermo Fisher Scientific). Cell supernatants were purified by protein A sepharose column as described (22). Purified antibodies were fluorescently labeled with Cy5.5 fluorophore as per the manufacturer's instructions (Sigma GEPA15604).

### Polymorphonuclear Leukocyte EV Generation

Polymorphonuclear leukocytes (PMNs) were isolated from healthy human volunteers by density gradient centrifugation and then stimulated with recombinant human TNFα for 20 min at 37°C to stimulate EV release (23). EVs were purified by differential centrifugation as described (20). For fluorescent observations, EVs were labeled using 50 μM of thiol reactive BODIPY FL maleimide or BODIPY TR dyes (Thermo Fisher Scientific, B10250, and D6116, respectively). Collection of blood samples from healthy human volunteers was approved by the East London and The City Local Research Ethics Committee (Rec Ref. 05/Q0603/34 ELCHA, London, UK). Informed consent was received from participants prior to inclusion in the study.

### ImageStream^*X*^ Analysis

All samples were acquired on an ImageStream^X^ MkII imaging cytometer, ×60 magnification; with low flow rate/high sensitivity using INSPIRE software as described (23). Isolated EVs were also analyzed using an NS300 Nanoparticle Tracker with a 488-nm scatter laser and high-sensitivity camera (Malvern Instruments Ltd., Malvern, UK). For each sample, particle scatter was recorded three times for 60 s each under flow conditions (arbitrary speed 50) at camera level 14 and analysis threshold 5, using the NTA 3.2 acquisition and analysis software.

### Enrichment of EV With Antibodies

A “phospholipid cake” was created by adding 100 μg of 1,2-dioleoyl-*sn*-glycero-3-phospho-l-serine (phospholipid, Avanti 181PS) to a pear-shaped glass flask and evaporating the chloroform solvent using a nitrogen stream. A solution of fluorescent antibody and fluorescent EV was prepared, using 10 μg of antibody for every 5 × 10^5^ EVs (using 1.5–2.5 × 10^6^ vesicles in a 0.5 ml reaction). The EV/antibody solution was added to the phospholipid cake. The solution was sonicated for 5 min on a setting of three amplitude microns using a Soniprep 150 ultrasonic disintegrator with an exponential probe. Enriched EVs were purified by differential centrifugation again as described to remove unbound antibody (20). Samples were then appropriately prepared for further experimentation.

### Enzyme-Linked Immunosorbent Assay (ELISA)

ELISA was performed by coating with 10 μg/ml of collagen II or ROS-modified collagen II (ROS-CII) or 10 μg/ml of recombinant mouse TNF (mTNF) as described (24). Antibody-enriched EVs were added as primary antibody in the ELISA, as well as anti-ROS-CII alone as a positive control and negative control antibody (10 μg per well). Binding was probed with anti-human IgG 1:1,000 dilution or anti-His Tag (Sigma Aldrich). TMB substrate was finally added to detect binding. The reaction was stopped using 50 μl of 20% sulfuric acid, and absorbance was read at 450 nm. All washes were performed using PBS—without calcium chloride and magnesium chloride.

### Immunofluorescence Staining of Arthritis Mouse Tissue

Sections of knee joints from antigen-induced arthritis (AIA) mice were deparaffinized, dehydrated, and air-dried, followed by fixation using 4% paraformaldehyde (PFA) in PBS. Antigen retrieval was performed by incubations in 0.02% HCl for 15 min at 37°C and 0.02% HCl and 15 mg/ml of pepsin solution for 45 min at 37°C. Tissue was then fixed again by incubating in 4% PFA solution before incubating in 50 mM of ammonium chloride to quench autofluorescence. Non-specific binding sites were blocked using 2.5% w/v powdered milk in PBS—for 1 h at room temperature. Added to the samples and incubated overnight in the dark at 4°C was 5 × 10^5^ BODIPY FL-labeled EV alone, 10 μg/ml of Cy5.5-labeled antibody-enriched EV, or 10 μg/ml of Cy5.5-labeled antibody alone. The following day, slides were washed in PBS and mounted using Fluoroshield with DAPI. Immunofluorescence staining was imaged on an Olympus BX61 microscope equipped with a Hamamatsu Orca-R2 digital camera. Images were acquired using Cell P/Sense software.

### *In vivo* Imaging

AIA was performed on female, 10-week-old C57BL/6 mice as described (25). Inflammation was stimulated in only one knee, with contralateral knees acting as controls. On day 22 and 24 h after stimulating inflammation in the right knee joint, mice were intravenously administered fluorescently labeled anti-ROS-CII (Cy5.5)-enriched EV (BODIPY TR) or EV alone (600,000 EVs for each injection). For IVIS Optical Imaging, mice were biofluorescently imaged as described (26). All experimental protocols were performed in compliance with the UK Animals (Scientific Procedures) Act 1986 regulations for the handling and use of laboratory animals (Home Office project license PPL no. 70/8264).

### Treated Knee Histology

For paraffin embedding, knee joints were harvested and incubated in 4% neutral buffered formalin followed by decalcification with EDTA for 3 weeks and embedding in paraffin wax. Knees were sectioned at 5 μM thickness with a minimum of two sections per knee used for scoring. Safranin O staining (Sigma, S8884) and hematoxylin-and-eosin (H&E, Sigma Aldrich, 102439) staining were performed as in (27). For collagen type X and anti-ROS-CII, immunostaining was done as above (28).

### Confocal Microscopy

Cryosectioning was done as described (29). Harvested knee joints were snap-frozen in OCT medium and followed by 17-μM-thick tissue sections. Knee cryosections were dried and mounted using a Fluoroshield mounting medium (Sigma, F6182) before being imaged on a Leica SP8 confocal microscope with the use of a 20× water-dipping objective. Images were attained with the use of sequential scanning of different channels at an XY resolution of 1,024 × 600 with a speed of 700 Hz, zoom factor of 1.28, and line average of 4. Images were analyzed with the image processing software IMARIS. Fluorescent localization of treatment was quantified using Image J/Fiji software. Cartilage regions of interest were drawn, and integrated density of both BODIPY TR and Cy5.5 was measured.

### Quantitative Real-Time Polymerase Chain Reaction

Total RNA was extracted from snap-frozen knee joints using the TRIzol method, as previously described (30). Reverse transcription of 1 μg of total RNA was done using a High-Capacity cDNA Reverse Transcription Kit (Thermo Fisher Scientific) with random primers and following manufacturer's protocol. Real-time PCR was carried out using power SYBR Green Master Mix in a real-time PCR system (Applied Biosystems Inc.). Data are expressed as relative units calculated by 2^−ΔΔCt^ by normalization relative to RPL32 and to fold change over naive control samples.

### Statistics

Statistical analysis was performed using GraphPad Prism 8 software and R 3.5.2. We have used several statistical measures appropriate to the experiments. We performed a mixed-model ANOVA (when the data were collected and analyzed together), allowing for a random intercept and slope of day or a two-way repeated-measures ANOVA with Holm–Sidak *post-hoc*, with Tukey *post-hoc* tests comparing the main effects of treatment. We also performed separate Kruskal–Wallis with Dunn *post-hoc* tests. For the localization experiment, we used R to model the Cy5.5 and BODIPY TR data with a separate three-way mixed-model ANOVA, using time, treatment, and knee as independent variables.

## Results

### Antibodies Incorporated Onto EV Retain Binding Specificity

**Figure 1:**
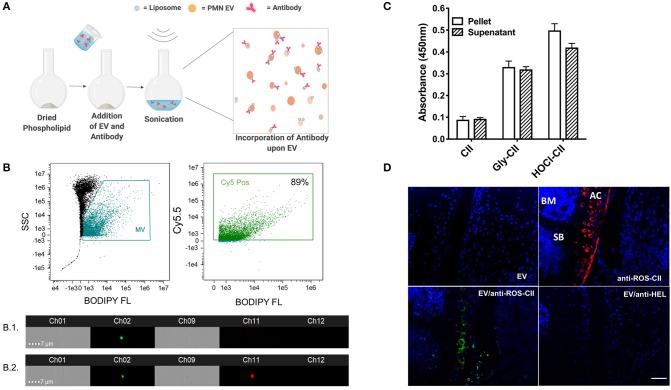
EV/anti-ROS-CII *in vitro* characterization. **(A)** Anti-ROS-CII antibodies were loaded onto PMN-derived EV (EV/anti-ROS-CII) through a process of energy dissemination and fusion with lipid vesicles, as outlined in the schematic created with BioRender. **(B)** Incorporation of anti-ROS-CII antibodies upon EV was assessed by ImageStream^X^. Representative image of EV (B.1) and EV enriched with anti-ROS-CII antibody (B.2) obtained from the ImageStream analysis, with bright field shown in channels 1 and 9, Ch01, and Ch09; side scatter in channel 12, Ch12; EV fluorescence (BODIPY) in channel 2, Ch02; and antibody fluorescence (Cy5.5) in channel 11, Ch11. Of the EV population, 89% was positive for the anti-ROS-CII fluorescence. **(C)** Antibody functionality after incorporation upon EV was tested by ELISA. Binding characteristics to native collagen type II (CII) and ROS modified CII (CII modified by ribose; Gly-CII and HOCl; HOCl-CII) were determined. Both pellet and removed supernatant were analyzed to determine the relative antibody reactivity loaded upon EV, as compared to the unbound. Data are shown as mean ± SEM, *n* = 3. There was a significant difference between reactivity to native CII vs. ROS-CII (Gly-CII and HOCl-CII, *p* < 0.001), but no main effect between pellet and supernatant (*p* > 0.05). **(D)** Binding specificity was also determined using sections from mouse antigen-induced arthritis knee tissue taken 7 days after induction. Immunofluorescence of EV (BODIPY FL) and EV enriched with either anti-ROS-CII (EV/anti-ROS-CII, Cy5.5) or control antibody specific to hen egg lysozyme (EV/anti-HEL) binding within the arthritic cartilage was assessed. Specific binding to articular cartilage (AC) was observed by anti-ROS-CII and EV/anti-ROS-CII but not with EV or EV/anti-HEL. SB, subchondral bone; BM, bone marrow. Scale bar = 50 μm.

EVs were isolated from PMN and analyzed for size and concentration by nanoparticle tracker analysis (Supplementary Figures 1A–C). Antibodies to ROS-CII were incorporated onto EV using aqueous energy dissemination, in which sonication results in the production of liposomes, which entrap the antibody while simultaneously integrating within the vesicle membrane (Figure 1A). For monitoring antibody incorporation, EV and anti-ROS-CII were fluorescently labeled with BODIPY FL and Cy5.5, respectively, and analyzed using ImageStream^X^. The EV population was gated through their characteristic low side scatter and positive BODIPY FL fluorescence and then probed for antibody fluorescence positivity (Cy5.5). Of the EV event population, 89% were positive for the anti-ROS-CII fluorescence, with a mean incorporation of 87% out of repeated and distinct preparations. ImageStream analysis of EV alone (B1) compare to EV enriched with anti-ROS-CII antibody (B2) showed that both EV and enriched EV are round with no structural disturbance (Figure 1B, Supplementary Figures 1E,F).

Following incorporation, EVs were pelleted and resuspended, and supernatants were removed to take any unincorporated antibody. Both resuspended pellet and supernatant were subjected to ELISA to determine presence of anti-ROS-CII in the fortified EV (EV/anti-ROS-CII). Specific binding of EV/anti-ROS-CII to ROS-CII was quantified, and a similar signal was detected in the supernatant, indicating ~50% antibody incorporation on the EV (Figure 1C). Immunofluorescence analysis was conducted to confirm the specific binding of EV/anti-ROS-CII to arthritic cartilage. Sections incubated with EV alone or EV fortified with isotype control antibody specific to hen egg lysozyme (EV/anti-HEL) exhibited minimal fluorescence. As expected, robust staining of damaged cartilage was observed using anti-ROS-CII. Importantly, following anti-ROS-CII incorporation onto EV, enhanced EV fluorescence was observed specifically within the arthritic cartilage (Figure 1D). Thus, we incorporated anti-ROS-CII antibody on EV, and these new structures display specific binding, which is augmented when compared to application of the single tool.

### EV Fortified With Antibodies to ROS-CII Localize to Arthritic Joints *in vivo*

**Figure 2:**
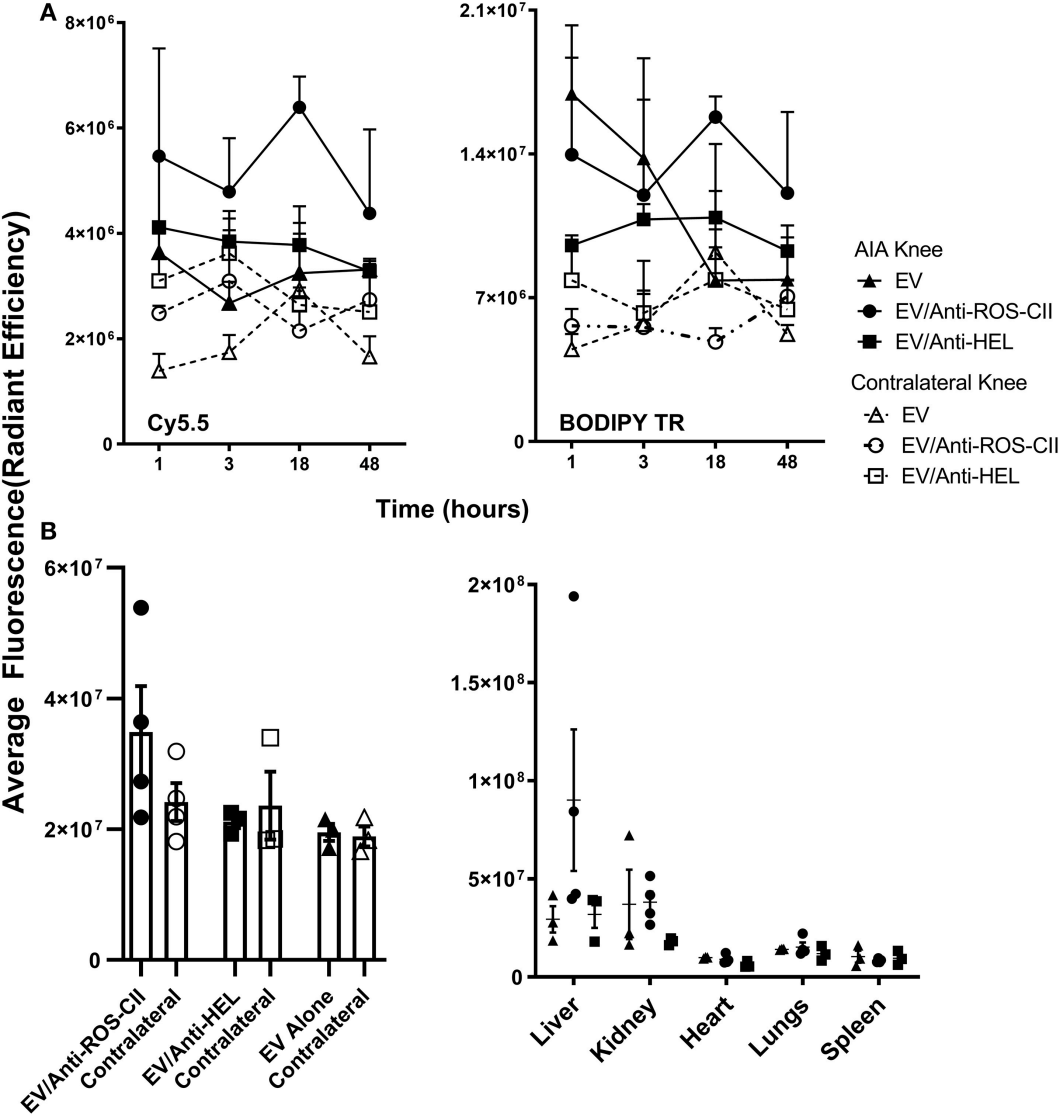
*In vivo* localization of EV/anti-ROS-CII. Antigen-induced arthritis was induced in the right knee of female C57BL/6 mice. One day after the stimulation of inflammation in the right knee, mice were injected with 6 × 10^5^ i.v. of either EV alone or EV/anti-ROS-CII or EV/anti-HEL. Localization of EV in the arthritic and contralateral knees was assessed by IVIS Optical Imaging. **(A)** A specific localization of EV/anti-ROS-CII in the arthritic knee was observed, with peak localization at 18 h post-administration in both the Cy5.5 and BODIPY TR channels, indicating antibody and EV co-localization (*p* = 0.009 for Cy5.5 fluorescence and *p* = 0.034 for BODIPY TR). **(B)** Knees and organs were imaged by IVIS in the BODIPY TR channel *ex vivo* at the end point of the experiment (48 h). Mice injected with EV/anti-ROS-CII showed higher retention of fluorescence signal within the arthritic knees compared to those treated with EV alone and EV/anti-HEL-treated samples (*p* < 0.05). Data are shown as mean ± SEM, *n* = 3 or 4 mice per group. Three-way mixed-model ANOVA was performed, with time, treatment, and knee as independent variables (the intercept and slopes of time and knee were allowed to vary for each mouse) with Holm–Sidak *post-hoc* tests of simple effects.

A mouse model of AIA was utilized to demonstrate joint-specific targeting of these scaffolds *in vivo*. Using IVIS optical imaging, we could visualize accumulation of BODIPY TR-labeled EV and Cy5.5-labeled antibody to the arthritic knee joint as compared with the healthy contralateral control knee. This specific localization of EV/anti-ROS-CII in the arthritic knee peaked at 18 h post-administration as quantified for both the Cy5.5 and BODIPY TR channels, indicating co-localization of the antibody with the EV (Figure 2A). A significantly lower signal was detected in the contralateral control knee as seen for both BODIPY TR fluorescence (*p* = 0.034) and Cy5.5 fluorescence (*p* = 0.009) at 18 h. Notably, the BODIPY TR signal for EV alone dropped dramatically from the early time points, indicating clearance or loss of retention of the vesicles within the joint tissue.

At the end-point of the experiment (48 h), knees and organs were harvested and imaged *ex vivo*. Mice injected with EV/anti-ROS-CII showed higher retention of EV fluorescence signal within the arthritic knees compared to EV alone (*p* < 0.05) and EV/anti-HEL (*p* < 0.05, Figure 2B). No off-target signal was detected systemically, except for some signal in the liver and kidney, likely reflecting antibody/EV clearance (Figure 2B).

### Enrichment of EV With Multiple Therapeutics

**Figure 3:**
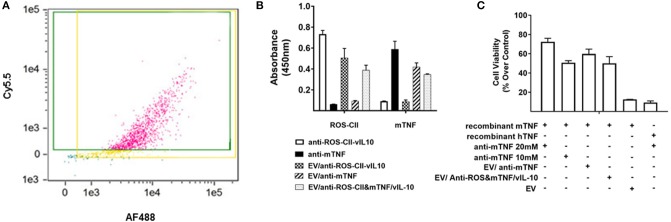
*In vitro* characterization of EV/anti-ROS-CII enriched with vIL10 and anti-mTNF. Anti-ROS-CII-vIL10 and anti-mTNF were loaded upon PMN EV through fusion with lipid vesicles. **(A)** Incorporation of anti-ROS-CII-vIL10 labeled with AF488 and anti-mTNF labeled with Cy5.5 upon EV was validated by ImageStream^X^. Incorporation of both antibodies resulted in 78% of EV staining double positive for both the anti-ROS-CII-vIL10 antibody and the anti-mTNF antibody. **(B)** ELISA plates were coated with recombinant mTNF or ROS-CII to test the binding specificity of the antibodies after EV incorporation. Both anti-ROS-CII and anti-mTNF embedded on the EV retained their specific binding to ROS-CII and mTNF, respectively. **(C)** The efficacy of anti-mTNF-enriched EV to inhibit mouse recombinant TNF-induced cell death in L929 cells was tested by MTT assay. Inhibition of cell death was evident only in EV/anti-mTNF and EV/anti-ROS-CII&mTNF/vIL-10. L929 cell death induced by human TNF (hTNF) was not inhibited by anti-mTNF. Data shown as mean ± SEM of *n* = 3, *p* < 0.0001. Data were analyzed by one-way ANOVA with Tukey *post-hoc* test.

EVs were fortified with either or both AF488-labeled anti-ROS-CII/vIL-10 and Cy5.5-labeled anti-mouse TNFα (anti-mTNF). Fortified EVs were analyzed for size by nanoparticle tracker analysis (Supplementary Figure 1D). ImageStream^X^ confirmed the presence of both proteins upon the EV following aqueous energy dissemination with ~80% EVs being positive for both antibody fluorophores (Figure 3A). EVs enriched with multiple bioactive payloads are round with no structural disturbance (Supplementary Figure 1F). Specific binding of antibody-enriched EV to ROS-CII and mTNF was determined using ELISA. Positive antibody controls (anti-ROS-CII-vIL10 and anti-mTNF) exhibited robust binding to their respective antigens. Importantly, EV loaded with both anti-ROS-CII-vIL-10 and anti-mouse TNFα antibodies retained binding to both murine TNF (mTNF) and ROS-CII (Figure 3B), indicating that that vesicle was amenable to be loaded with more than one therapeutic agent. Of importance is that not only can the EV afford a scaffold of inert anti-inflammatory properties, but it can also cargo bioactive payloads. To address this, a functional bioassay for mTNF was performed using L929 cell survival. Following application of a lethal concentration of murine recombinant TNF, soluble anti-mTNF, but not EV alone, inhibited cell death in a concentration-dependent manner. In contrast, vesicles enriched with anti-mTNF antibody retained the ability to inhibit cell death similarly to anti-mTNF antibody alone. Anti-mTNF antibody was ineffective when recombinant human TNF was used in the cell culture medium instead of murine TNF (Figure 3C), indicating species specificity.

### EV/Anti-ROS-CII Loaded With Therapeutics Accelerates Resolution of Joint Inflammation

**Figure 4:**
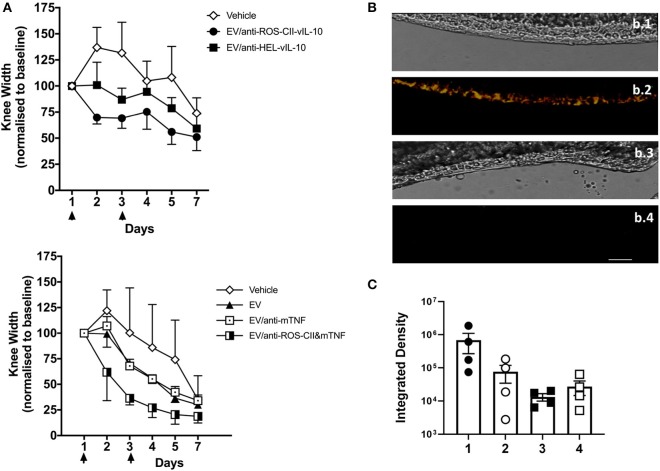
Treatment of antigen-induced arthritis with EV enriched with monotherapy. Antigen-induced arthritis was induced in the right knee of female C57BL/6 mice. On day 1 and 3, mice were injected with 6 × 10^5^ i.v. of EV, EV/anti-ROS-CII-vIL-10, EV/anti-HEL-vIL-10, EV/anti-mTNF, EV/anti-ROS-CII&mTNF, or PBS vehicle control. **(A)** Treatment points are indicated by black arrows. Knee swelling was quantified daily using digital calipers. Data are expressed as mean ± SEM, *n* = 5–8 mice per treatment group and *n* = 3–6 mice for vehicle group. Top panel were analyzed by two-way repeated-measures ANOVA (with time and treatment as independent variables) with Holm–Sidak *post-hoc* tests. As the data were all normalized to day 1, day 1 was omitted from the analysis (as the values are all 100). A significant difference between vehicle and EV/anti-ROS-CII-vIL-10 (*p* = 0.028) was observed. Lower panels show analysis by a mixed-model ANOVA, allowing for a random intercept and slope of day (which was entered as a continuous variable), with treatment as a fixed factor. There were no significant differences between the vehicle group and the EV alone (*p* = 0.216) or EV/anti-TNF (*p* = 0.216). A significant difference between vehicle and EV/anti-ROS&mTNF (*p* = 0.013) was observed. **(B)** Knees from EV/anti-ROS-CII-vIL10- and EV/anti-HEL-vIL-10-treated mice were sectioned and imaged by confocal microscopy to identify EV/antibody localization; bright-field images were also taken for reference (b.1, b.3). EV/anti-ROS-CII-vIL10 AIA knee (b.1, b.2); EV/anti-HEL-vIL-10 AIA knee (b.3, b.4). Red fluorescence: BODIPY TR (EV); cyan fluorescence: Cy5.5 (anti-ROS-CII); yellow fluorescence in b.2 indicates co-localization of anti-ROS-CII antibody and EV. Scale bar = 40 μm. **(C)** Quantification of BODIPY TR and Cy5.5 fluorescence in the cartilage of knee joint sections. 1: EV/anti-ROS-CII-vIL-10 AIA knee, 2: EV/anti-ROS-CII-vIL-10 contralateral knee, 3: EV/anti-HEL-vIL-10 AIA knee, and 4: EV/anti-HEL-vIL-10 contralateral knee.

To test the efficacy of the EV to cargo *in vivo*, AIA mice were treated intravenously with EV/anti-ROS-CII-vIL-10 (on days 1 and 3) post-induction of arthritis. This EV/anti-ROS-CII-vIL-10 treatment afforded a higher degree of reduction in knee swelling (*p* = 0.028) and was more effective than EV enriched with vIL-10 fused to isotype control antibody—that is, specific to hen egg lysozyme (anti-HEL-vIL-10; Figure 4A). Similarly, while EV/anti-mTNF was associated with modest attenuation of joint inflammation similar to EV alone (*p* = 0.216), administration of EV/anti-ROS-CII&mTNF enhanced the reduction in arthritic knee swelling (*p* = 0.013).

**Figure 5:**
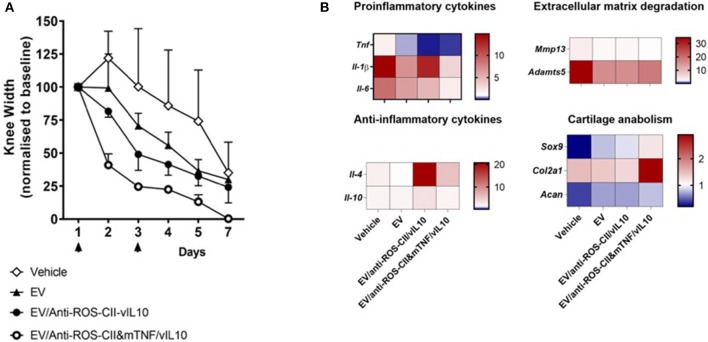
Treatment of antigen-induced arthritis with EV fortified with combined treatment. Antigen-induced arthritis was induced in the right knee of female C57BL/6 mice. On day 1 and 3, mice were injected with 6 × 10^5^ i.v. of EV alone, EV/anti-ROS-CII-vIL-10, EV/anti-ROS-CII&mTNF/vIL-10, or vehicle. **(A)** Knee swelling was monitored using digital calipers. Data are expressed as mean ± SEM, *n* = 5 mice per treatment group (*n* = 3 mice for the vehicle group). Data were analyzed using a mixed-model ANOVA, allowing for a random intercept and slope of day (which was entered as a continuous variable), with treatment as a fixed factor. There were significant main effects of treatment and day, and Holm–Sidak *post-hoc* tests comparing the overall knee width of each treatment group to the vehicle group indicated lower knee widths for EV/anti-ROS-CII-vIL-10 (*p* = 0.0499) and EV/anti-ROS-CII-vIL-10/anti-TNF (*p* = 0.001) treatment groups (control groups are the same as in Figure 4A, bottom panel). There were no significant differences between the vehicle group and the EV alone (*p* = 0.216). **(B)** Local gene expression was measured by quantitative PCR (qPCR) in the arthritic joints at day 7. Data were normalized over the *RPL32* housekeeping gene and are expressed as fold change over control knee joints from naive mice, with average expression levels represented using a heat map (*n* = 3 mice per group).

At end-point, mouse joints were harvested, snap-frozen, and sectioned prior to imaging to visualize EV and anti-ROS-CII-vIL-10 retention in the arthritic cartilage (Figure 4B). Both EV fluorescence (BODIPY TR) and anti-ROS-CII fluorescence (Cy5.5) could be detected by confocal microscopy within the joint cartilage of mice treated with EV/anti-ROS-CII-vIL-10 (yellow overlay; Figure 4B, b.2). This was exclusive to the ipsilateral, inflamed knee, with cartilage treated with control EV/anti-HEL-vIL10 showing no specific fluorescent localization (Figure 4B, b.4). Quantification of the EV and antibody fluorescence in the cartilage of AIA and contralateral joints confirmed this (Figure 4C). Importantly, knee swelling in AIA mice treated with EV/anti-ROS-CII&mTNF/vIL-10 combination therapy resolved significantly faster, returning to baseline levels compared to EV alone (*p* = 0.001). Treatment with single-cargo EVs, anti-ROS-CII-vIL-10 therapy, also accelerates edema reduction compared to vehicle (*p* = 0.049) but was slower than treatment with EV/anti-ROS-CII&mTNF/vIL-10 (Figure 5A).

### Comparative Gene Expression Analysis in the Knee Joints

To elucidate potential mechanisms behind the anti-arthritic effects of fortified EV, gene expression was measured in the arthritic joints at day 7. Mice treated with EV/anti-ROS-CII-vIL-10 or EV/anti-ROS-CII&mTNF/vIL-10 exhibited reduced expression of (a) pro-inflammatory gene products like *Tnf*, *Il-1*β, and *Il-6* and (b) *Mmp13* and *Adamts5* genes involved in cartilage degradation. In contrast, increased expression of (i) anti-inflammatory cytokine gene product *Il-4* and, to a lesser extent, *Il-10* and (ii) *Sox9, Acan*, and *Col2a1* genes, responsible for cartilage maintenance and/or regeneration, was observed (Figure 5B).

### Histological Analysis of the Knee Joints

**Figure 6:**
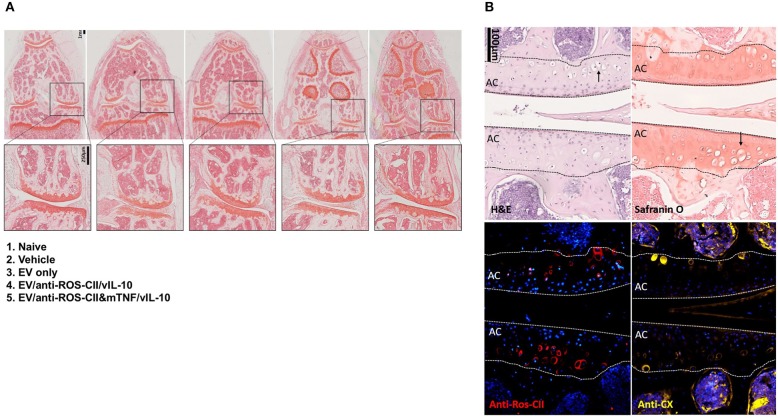
Histological analysis of excised knee joints. Histopathology analysis of knee sections revealed a spectrum of anti-inflammatory responses. **(A)** Safranin O staining of example knees comparing vehicle to treatment groups which were improved to various degrees. We observed improved histological arthropathy phenotype with mice treated with EV enriched with both vIL10 and anti-TNFα which in some mice as shown in this example resemble naive phenotype. Safranin O staining of cartilage from non-treated AIA knee (vehicle) displayed weaker staining and abundant chondrocyte death. The cartilage from healthy control animals was smooth, with clear strong staining with safranin O. In the EV-treated knee, we observed areas of weak safranin O staining within the cartilage layers. **(B)** Examples from other groups of sections within the central region of the knee were stained with hematoxylin and eosin (H&E), safranin O, anti-ROS-CII, and anti-collagen type X. Example images show corresponding regions within the same diseased knee in the anti-ROS-CII&mTNF/vIL-10 treatment group. Chondrocyte death within the cartilage can be seen (black arrows). The presence of damaged cartilage is stained by anti-ROS-CII (oxidative post-translationally modified collagen type II neo-epitope, red). These areas corresponded with regions of chondrocyte hypertrophy indicated by staining with antibody to collagen type X (anti-CX, yellow). Lines define the boundaries of the articular cartilage (AC).

Histopathology scoring was performed on safranin O- and H&E-stained slides, while immunohistochemistry analyses were conducted for anti-ROS-CII and anti-collagen type X antibodies. An example of each detailed scoring is shown in Supplementary Figure 2. Compared to vehicle and EV comparator groups, EV delivery of IL-10 or anti-TNFα was associated with reduced joint inflammation and chondrocyte death. While we did not observe a significant difference in cartilage matrix integrity and safranin O staining, we did observe a reduction in pannus formation, synovial fluid immune cells, chondrocyte death, and bone marrow involvement. This cumulative analysis of synovial inflammation and chondrocyte death is displayed in Supplementary Figure 2. In the non-treated control group, there was no resolution of synovial inflammation. In the EV/anti-ROS-CII&mTNF/vIL-10 group of mice, clinical signs of arthropathy were abolished over time with associated clear indications of a reduction in the histological signs of joint inflammation. In some mice, we observed complete resolution (Figure 6A), but we mostly observed a range of histological improvement. The data indicated that knee swelling changes preceded signs of histological improvement. Compared to single treatments, the double-cargo EVs afforded a higher degree of protection on a variety of histological markers, with particular efficacy in reducing chondrocyte death. Immunostaining with anti-ROS-CII revealed presence of areas of mixed residual damaged cartilage and repair—which corresponded with regions of chondrocyte hypertrophy—indicated by staining with antibody to collagen type X (Figure 6B).

## Discussion

In this study, we describe a proof-of-concept study using a specific anti-ROS-CII antibody delivery system affording anatomically specific targeting of an EV and EV-packaged bioactive payload to the inflamed joint. Our principal aims were to demonstrate fidelity of anatomical targeting using this technology, to establish some key pharmacokinetic outcomes, and to show that targeting bioactive payloads following systemic administration was associated with a therapeutic benefit. Herein, we have harnessed two distinct biological features: one represented by a targeting antibody specific for arthritic cartilage (anti-ROS-CII) and the other being packaged reparative information delivered by PMN EV. Moreover, we constructed and validated a new therapeutic tool that combines these benefits. Our aim was to define whether selective targeting could achieve an improved pharmacodynamic profile. Indeed, using a model of joint arthritis in mice, we could demonstrate that enriching EV with anti-ROS-CII (a) targets EV to arthritic joints following intravenous injection; (b) enhances binding and retention of EV within the arthritic cartilage; and (c) accelerates the attenuation of clinical and synovial inflammation following fortification with anti-inflammatory drugs.

Plasma membrane-shed vesicles complement intercellular communication driven by soluble mediators, like cytokines, autacoids, and hormones. PMN EVs hold powerful anti-inflammatory and pro-resolving qualities and can penetrate >100 μm deep into the cartilage matrix (15). Moreover, the inherent anti-inflammatory effect exhibited by PMN EV has been exploited by loading additional proteins or lipid mediators, to enhance innate immune cell function to expedite resolution responses (18, 19). A recent study has gone a step further and fused PMN membranes onto polymeric cores, showing that these EV/polymeric hybrids can neutralize pro-inflammatory cytokines, suppress synovial inflammation, and penetrate cartilage while providing chondroprotection against joint damage. The EV/polymeric hybrid core demonstrated a significant therapeutic effect in a mouse model of collagen-induced arthritis and in a human transgenic mouse model of arthritis (21). Nevertheless, efficacy was observed only following local administration, and similar observations were made by us too with natural EVs (20, 23). Obviously, a delivery modality which can be introduced systemically has greater flexibility for translational studies in humans.

To target EVs to the arthritic joint following systemic treatment, we harness our own discovery of an antibody to disease-modified CII. Indeed, inflammation leads to the generation and release of large amounts of enzymes and reactive oxidants (e.g., O2^−^, NO^·^, H_2_O_2_, or HOCl) which provoke post-translational modification of the extracellular matrix and other proteins exposed on the cellular plasma membrane. We have produced and characterized an antibody that is specific to CII post-translationally modified by oxidants (anti-ROS-CII). This unique antibody has been validated in a series of studies demonstrating strong immunostaining on arthritic but not healthy cartilage, specific targeting to arthritic joint *in vivo*, and attenuation of knee inflammation by drug targeted by anti-ROS-CII (28, 31). Once grafted onto EV, the anti-ROS-CII antibody acted as an arthritic cartilage-specific navigator, leading to an accumulation of EV selectively to arthritic joints with a peak at 18 h post-i.v. injection (Figure 2). This was in clear contrast to EV alone that could be found in the tissue (probably through passive extravasation or other unknown mechanisms of trafficking) but evidently were not retained in the joint since they were almost absent at 18 h post-injection. EV/anti-ROS-CII signal was not detected in the heart, lungs, or spleen, but some degree of clearance through the liver and kidney was detected, as expected for vesicles that use the reticulum–endoplasmic pathway for elimination, a path used also by apoptotic bodies and other foreign bodies.

This favorable pharmacokinetic profile translated into local pharmacodynamic improvement, consistent with a model of depot release of payload into the local joint microenvironment, thus raising the possibility that targeted EV could be used to cargo specific therapies. This technology would not only have the potential to augment efficacy but could and should also markedly improve safety by limiting systemic exposure. A major drawback in the use of biologics is not only their costs and proportion of patient unresponsiveness (32); there is also a major medical issue that is the induction of immune suppression with re-emergence of silent and symbiotic infections, on top of host-to-drug antibody generation (33). As such, there is an urgent need to harness the positive properties of biologics while avoiding the burden of important side effects. We reasoned that our EV/anti-ROS-CII technology could be of use here and have tested the pharmacodynamic improvement of targeted anti-mTNF and vIL-10 and provide proof of concept for further optimization and development.

EV enriched with anti-ROS-CII fused to an anti-inflammatory cytokine, vIL-10, or anti-mTNF accelerated reduction of knee swelling compared to EV loaded with control anti-hen egg lysozyme fused to vIL-10 or with anti-TNF only (Figure 4). In a separate set of experiments, we enriched the EV with two therapeutic agents: the anti-inflammatory cytokine, vIL-10, and a pro-inflammatory cytokine blocker, and the anti-mTNF antibody. Combined EV/anti-ROS-CII targeted treatment of anti-mTNF and vIL-10 resulted in accelerated resolution of knee swelling, as compared to a single vIL-10 treatment (Figure 5A). This clinical outcome is supported by evidence of improvement in synovial histopathology. The knee swelling effects, however, were more marked and less variable than the efficacy effects observed histologically—a differential entirely consistent with the differing kinetics of resolution of knee edema compared to resolution of the cellular inflammatory response. In terms of anatomical localization within the knee capsule, in some of the sections, we observed residual staining with anti-ROS-CII within the chondrocytes localized in the intermediate and deep cartilage zones. Interestingly, in the same region, there appeared to be enhanced staining on hypertrophic chondrocytes, an observation which related to retraction of the tide mark as evidenced by safranin O and increased collagen X staining. These results offer presumptive evidence for a chondroprotective effect. Further studies in a more chronic setting are required to delineate the exact phenotype of this response and to establish the relationship between enhanced staining of this putative immature chondrocyte and the dynamics of cartilage repair.

To gain some mechanistic insight for the added value provided by this new technology, we monitored a variety of gene products by qPCR analysis. The results indicated a degree of cartilage remodeling and repair which was incited by the combined (EV/anti-ROS-CII&mTNF/vIL-10) therapy over and above that attained by the single therapeutic approaches (EV alone or anti-ROS-CII/vIL-10). Augmented expression of anabolic genes like *Acan, Sox9*, and *Col2a2* was quantified following EV/anti-ROS-CII&mTNF/vIL-10 with lower degradative genes *Mmp13* and *Adamts5*. The anabolic effect can be due to inherent variety of EV mediators including annexin A1 (20) and/or pro-resolving lipid mediators like resolvin D1 (34). This genomic response complemented the morphological evidence that there was reduced chondrocyte loss. The ability of EV to penetrate into the cartilage plate gives confidence that chondroprotection within the superficial and intermediate cartilage zones is a viable therapeutic goal (20). These remodeling effects of EV/anti-ROS-CII&mTNF/vIL-10 were complemented by different expressions of inflammatory gene products: pro-inflammatory *Tnf*, *Il-1*β, and *Il-6* expression was lower, while anti-inflammatory *Il-4* was higher in treated mice. These readouts were in partial agreement with macroscopic and histological analyses and could underpin some of the effects on cartilage, considering the central role that IL-1β plays in chondrocyte reactivity (35).

Histopathology analysis of knee sections revealed an important outcome with respect to anti-ROS-CII binding, which was detected in the lacunae of a range of chondrocyte forms and was localized in the intermediate and deep cartilage. Interestingly, there appeared to be enhanced staining on hypertrophic chondrocytes, an observation which related to retraction of the tide mark as evidenced by safranin O and increased collagen X staining. There was a degree of discrepancy between the diverse profiles of histopathology compared to unique observed knee edema resolution: these differences probably reflect differences in the resolution of the vascular response compared to a slower kinetics for resolution of cellular inflammation and synovial hypercellularity. The totality of the genomic, clinical, and histological outputs support the concept of a viable anti-inflammatory signal originating from the targeted cartilage plate. Obviously, this model format was designed to demonstrate proof of concept of active payload delivery and not optimized or powered for the differing kinetics of gene, edema, synovitis, and cartilage attenuation and remodeling. A key focus of further work will be to explore this mechanism in the context of chondroprotection.

In summary, our new technology enables a simple, cost-effective, and reproducible approach for enriching EV with a variety of therapeutics, offering anatomical targeting of anti-inflammatory payloads to an inflamed joint. We have demonstrated a concordance between effective delivery and key efficacy outcomes and gained insight into the potential for more optimized delivery modalities to deliver chondroprotective effects in a more chronic setting. The linchpin of our therapeutic strategy is the potential to mix therapeutics that are loaded upon the EV. With a personalized medicine approach, alongside the successful systemic targeting of therapeutics directly to the arthritic joint, greater treatment efficacy with fewer side effects can be achieved.

## Data Availability Statement

The datasets generated for this study are available on request to the corresponding author.

## Ethics Statement

Collection of blood samples from healthy human volunteers was approved by the East London and the City Local Research Ethics Committee (Rec Ref. 05/Q0603/34 ELCHA, London, United Kingdom). Informed consent was received from participants prior to inclusion in the study. The patients/participants provided their written informed consent to participate in this study. The animal study was reviewed and approved by UK Home Office. All experimental protocols were performed in compliance with the UK Animals (Scientific Procedures) Act 1986 regulations for the handling and use of laboratory animals (Home Office project license PPL no: 70/8264).

## Author Contributions

AN and HA planned the project. AN, LT, and MP designed the experiments. LT, BT, HR, MF, MS, CV, and M-BV performed and analyzed *in vitro* experiments. LT, JT, LN, and HL performed and analyzed *in vivo* experiments. LT and AN wrote the manuscript. All other authors provided advice and oversight of the manuscript.

### Conflict of Interest

MF is a self-employed consultant in experimental pathology. He has no commercial or financial interests that could be construed as a potential conflict of interest. The remaining authors declare that the research was conducted in the absence of any commercial or financial relationships that could be construed as a potential conflict of interest.
