# Supplementary material for: Targeting Extracellular Vesicles to the Arthritic Joint Using a Damaged Cartilage-Specific Antibody
Source: Front Immunol. 2020 Feb 14;11:10. doi: 10.3389/fimmu.2020.00010 (PMC7033748; doi:10.3389/fimmu.2020.00010)
Supplement: Supplementary file 1 [file Data_Sheet_1.pdf]

Supplementary Material

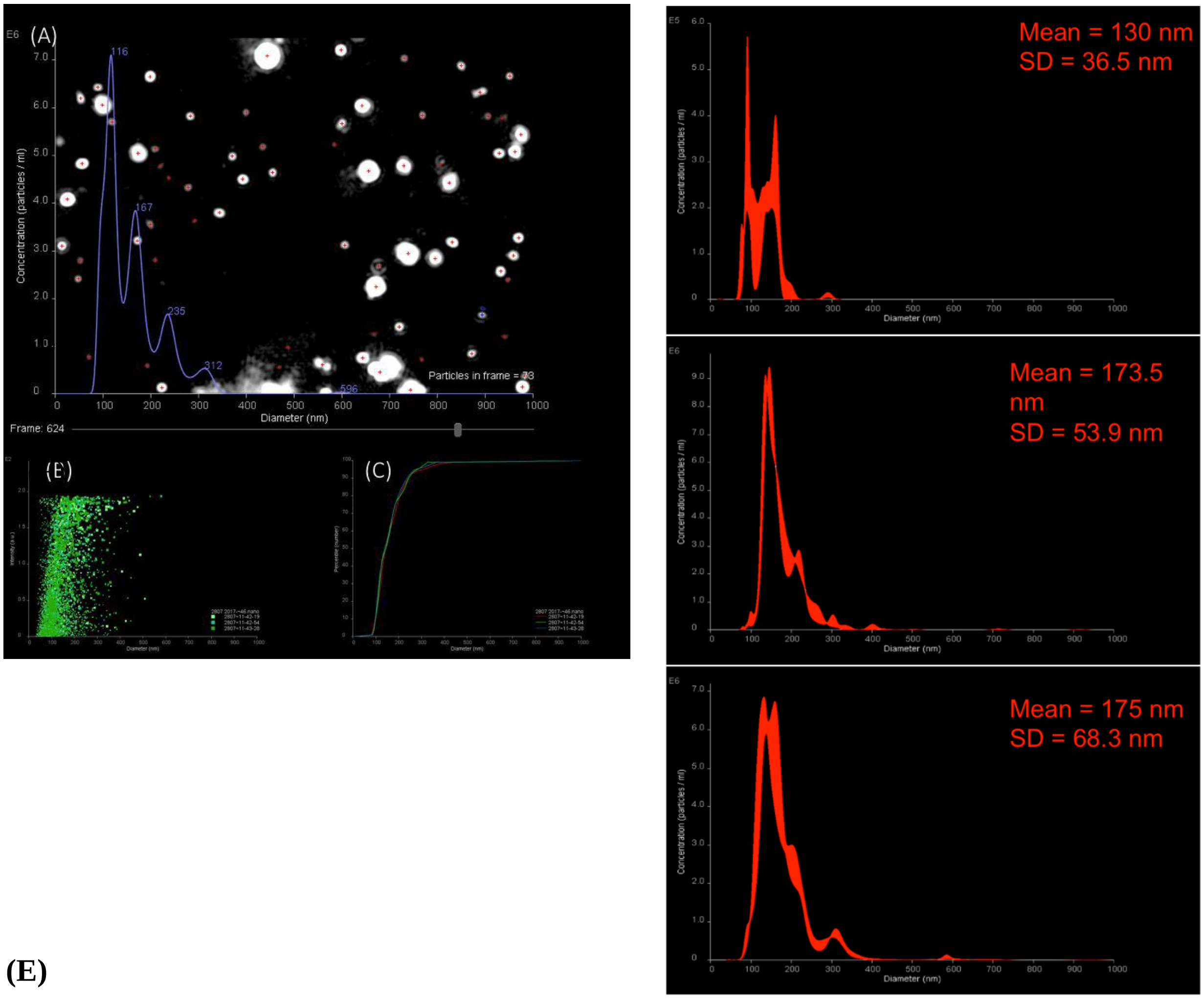

(E)

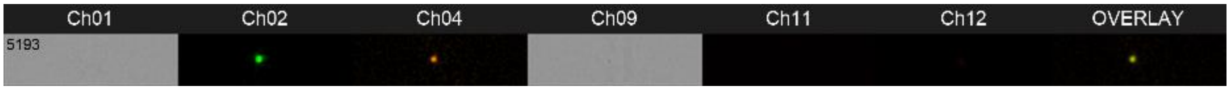

(F)

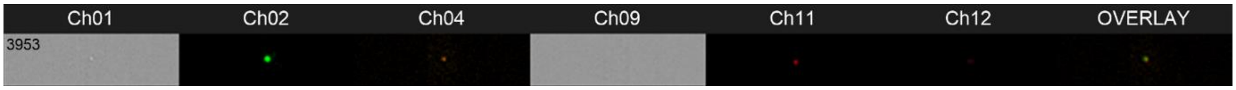

Supplementary Figure 1. Representative characterization of EV alone and EV enriched with antibodies. PMN EV from healthy donors were isolated and analyzed using nanoparticle tracking (A-C). Plot showing the distribution of size in nm against concentration of the isolated sample (A), scatter plot showing the size and intensity of all events run through the analyzer (B) and cumulative plot showing the percentile of the population and size in nm (C). The mean size of PMN derived EV from healthy donors or enriched with a single antibody (anti-ROS-CII) or with multiple antibodies (anti-

ROS-CII&TNF) (D). EV population show the expected average size, when loaded with treatment, size increases. Representative Imagestream[X] images of EV enriched with single antibody (E- Ch02 and Ch04 positive) or multiple antibodies (F- Ch02, Ch04 and Ch11 positive). Ch01 & Ch09; brightfield images, Ch02; AF488 fluorescence (anti-ROS-CII), Ch04; BODIPY TR fluorescence (EV), Ch11; Cy5 fluorescence (anti-TNF), Ch12; side scatter. n = 4 replicates.

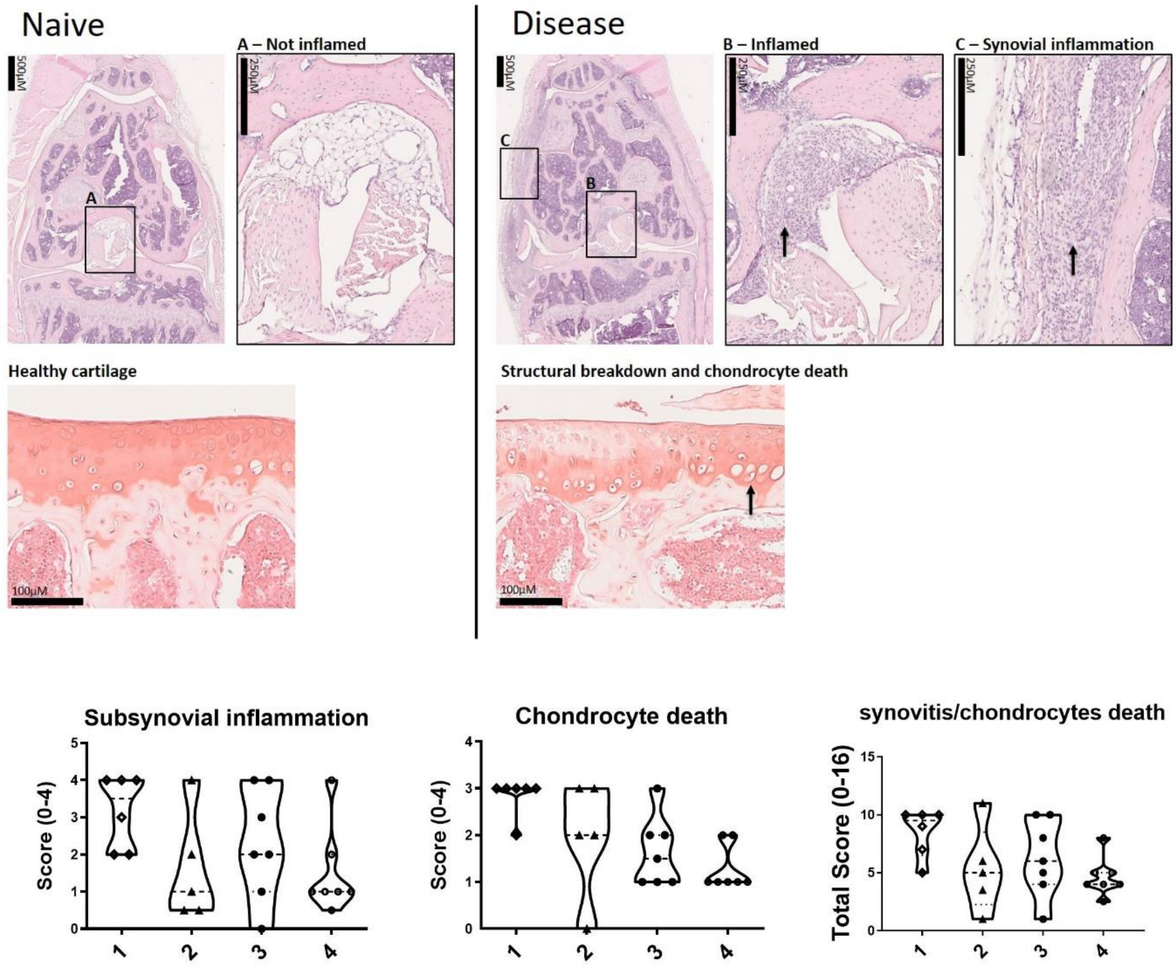

Supplementary Figure 2. Representation of histological scoring of knee joints. Naive mice show no evidence of inflammation within the joint. No inflammatory infiltrate (A), a healthy synovium without hyperplasia, and healthy, intact cartilage, depicted by strong safranin O staining. Diseased mouse knees show inflammation throughout joint tissues, with abundant infiltration of inflammatory cells (B, C) with synovial thickening (C), and loss of safranin O staining in the cartilage accompanied by chondrocyte death. (D) Scoring of sub-synovial inflammation, chondrocytes death and total composite synovitis and chondrocyte death score is shown. For every measure, separate Kruskal-Wallis tests were performed with Dunn *post-hoc* tests comparing all groups against control non-treated group. Significant differences were observed when comparing chondrocyte death: vehicle versus EV/anti-ROS-CII&mTNF/vIL-10 (p = 0.014). 1 is non treated group; 2 treated with EV only; 3 treated with EV/anti-ROS-CII/vIL-10 and 4 treated with EV/anti-ROS-CII&mTNF/vIL-10.
